# Dr. E. J. Fountain

**Published:** 1861-05

**Authors:** 


WE regret to announce the death of Dr. E. J. FOUNTAIN, which took place at Davenport, Iowa, on the 29th of March, in the thirty-third year of his age. Dr. Fountain's contributions to medical periodical literature were numerous and valuable; but he is best known by his researches of the medical uses of the chlorate of potassa, on which he wrote several highly interesting papers.

